# Treating activated regulatory T cells with pramipexole protects human dopaminergic neurons from 6‐OHDA‐induced degeneration

**DOI:** 10.1111/cns.14883

**Published:** 2024-08-04

**Authors:** Adrián Guevara‐Salinas, Citlalli Netzahualcoyotzi, Diana Denisse Álvarez‐Luquín, Erandi Pérez‐Figueroa, Edgar E. Sevilla‐Reyes, Carlos Castellanos‐Barba, Vera Teresa Vega‐Ángeles, Edgar Terán‐Dávila, Enrique Estudillo, Iván Velasco, Laura Adalid‐Peralta

**Affiliations:** ^1^ Laboratorio de Reprogramación Celular Instituto Nacional de Neurología y Neurocirugía “Manuel Velasco Suárez” Mexico City Mexico; ^2^ Instituto de Fisiología Celular – Neurociencias Universidad Nacional Autónoma de México Mexico City Mexico; ^3^ Centro de Investigación en Enfermedades Infecciosas Instituto Nacional de Enfermedades Respiratorias “Ismael Cosío Villegas” Mexico City Mexico; ^4^ Laboratorio de Transcriptómica e Inmunología Molecular Instituto Nacional de Enfermedades Respiratorias "Ismael Cosío Villegas" Mexico City Mexico; ^5^ Laboratorio Nacional de Citometría de Flujo Instituto de Investigaciones Biomédicas UNAM Mexico City Mexico

**Keywords:** 6‐hydroxydopamine, dopaminergic neurons, neuroprotection, Parkinson's disease, pramipexole, Tregs

## Abstract

**Background:**

Parkinson's disease (PD) is a chronic neurodegenerative disorder characterized by the loss of dopaminergic neurons in the *substantia nigra*, which promotes a sustained inflammatory environment in the central nervous system. Regulatory T cells (Tregs) play an important role in the control of inflammation and might play a neuroprotective role. Indeed, a decrease in Treg number and function has been reported in PD. In this context, pramipexole, a dopaminergic receptor agonist used to treat PD symptoms, has been shown to increase peripheral levels of Treg cells and improve their suppressive function. The aim of this work was to determine the effect of pramipexole on immunoregulatory Treg cells and its possible neuroprotective effect on human dopaminergic neurons differentiated from human embryonic stem cells.

**Methods:**

Treg cells were sorted from white blood cells of healthy human donors. Assays were performed with CD3/CD28‐activated and non‐activated Treg cells treated with pramipexole at concentrations of 2 or 200 ng/mL. These regulatory cells were co‐cultured with in vitro‐differentiated human dopaminergic neurons in a cytotoxicity assay with 6‐hydroxydopamine (6‐OHDA). The role of interleukin‐10 (IL‐10) was investigated by co‐culturing activated IL‐10‐producing Treg cells with neurons. To further investigate the effect of treatment on Tregs, gene expression in pramipexole‐treated, CD3/CD28‐activated Treg cells was determined by Fluidigm analysis.

**Results:**

Pramipexole‐treated CD3/CD28‐activated Treg cells showed significant protective effects on dopaminergic neurons when challenged with 6‐OHDA. Pramipexole‐treated activated Treg cells showed neuroprotective capacity through mechanisms involving IL‐10 release and the activation of genes associated with regulation and neuroprotection.

**Conclusion:**

Anti‐CD3/CD28‐activated Treg cells protect dopaminergic neurons against 6‐OHDA‐induced damage. In addition, activated, IL‐10‐producing, pramipexole‐treated Tregs also induced a neuroprotective effect, and the supernatants of these co‐cultures promoted axonal growth. Pramipexole‐treated, activated Tregs altered their gene expression in a concentration‐dependent manner, and enhanced TGFβ‐related dopamine receptor regulation and immune‐related pathways. These findings open new perspectives for the development of immunomodulatory therapies for the treatment of PD.

## Graphical abstract

Pramipexole‐treated, activated regulatory T cells protect dopaminergic neurons against 6 OHDA damage and promote primary neurite length. This could be due to the production of the regulatory cytokine IL‐10 and an increased expression of genes related to regulation and neuroprotection.

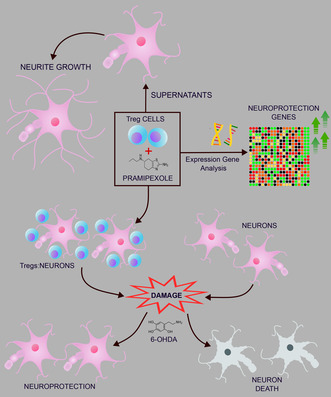

## 1 INTRODUCTION

Parkinson's disease (PD) is the second most common neurodegenerative disorder after Alzheimer's disease. It is characterized by the loss of dopaminergic neurons in the *substantia nigra pars compacta* (*SNpc*) and the appearance of alpha‐synuclein (α‐Syn) protein inclusions called Lewy bodies. The main motor symptoms are bradykinesia, rigidity, postural instability, and resting tremor.^1^, ^2^ PD affects 1%–2% of the population over 65 years of age, with a global incidence of 0.61 and a prevalence of 0.52 per 100,000 population in the period 1990–2019.^3^

Neuroinflammation, a key factor in PD, is caused by the interaction between central nervous system (CNS) cells and peripherally recruited immune cells.^4^ In the CNS, α‐Syn aggregates activate microglia, astrocytes, and immune cells, leading to increased production of proinflammatory cytokines such as interleukin‐6 (IL‐6), the tumor necrosis factor α (TNFα), and interleukin 1β (IL‐1β), as well as chemokines such as CCL2 and CXCL8. The shift of microglia to an inflammatory or M1 phenotype results in increased infiltration of both CD4+ and CD8+ T lymphocytes into the CNS, reaching the SNpc.^5^, ^6^ Increased production of reactive oxygen species (ROS) and reactive nitrogen species (NOS) is also observed due to the stress caused by neuronal death and mitochondrial damage. Together, this creates a cycle of activation and neuroinflammation that leads to neuronal degeneration and a persistent inflammatory environment.^4^, ^7^, ^8^

Among the various models that have been developed to study the damage to dopaminergic neurons in PD, cultures of dopaminergic neurons differentiated from human embryonic stem cells (hESC) offer the advantages of avoiding the interference of oncogenes characteristic of cell lines, and that the resulting mesencephalic dopaminergic neurons are capable of releasing dopamine and exhibit action potentials similar to functional dopaminergic neurons in the human brain.^9^, ^10^ hESC have been widely used to develop treatments and therapeutic strategies for PD.^11^

Dopamine agonists are used to treat PD, with levodopa (L‐dopa) being the gold standard; both types of compounds have been found to regulate the response of various immune cells by activating dopamine receptors (DRs). DRs are divided into two groups: D1‐type receptors, which include D1 and D5, and D2‐type receptors, including D2, D3, and D4.^12^, ^13^ D1‐type receptors are coupled to stimulatory G proteins, whereas D2‐type receptors are coupled to inhibitory G proteins.^13^

In particular, the dopaminergic agonist pramipexole has a high affinity for D2‐type receptors, especially D2 and D3, which reduces the adverse effects caused by non‐specific activation of other receptors. In addition, pramipexole has effects on the immune system, such as reducing the production of proinflammatory cytokines in lymph nodes.^14^, ^15^

On the other hand, the activation of D1 and D2 receptors in human CD8+ regulatory T cells (CD8+ CD28−) decreases their regulatory and cytotoxic capacity. A similar effect has been observed in CD4+ CD25^high^ regulatory T cells (Tregs), reducing IL‐10 production, although this effect is less pronounced in activated Treg cells.^16^, ^17^ Furthermore, activation of the D1 receptor on Tregs reduces their regulatory capacity, as well as the production of IL‐10 and the transforming growth factor (TGFβ).^17^

Tregs have been suggested to participate in neuroprotection by suppressing the effector response. Treg cell depletion has been reported to increase neuroinflammation in 1‐methyl‐4‐phenyl‐1,2,3,6‐tetrahydropyridine (MPTP)‐treated mouse models of PD.^7^ In addition, transfer of activated Treg cells to MPTP‐treated mice suppressed microglial activation and increased dopaminergic neuron survival.^18^

Although pramipexole is widely used for the symptomatic treatment of PD, little is known about the stimulatory effect of this dopaminergic agonist on immunoregulatory cells. In particular, Treg cells have shown promising neuroprotective effects in mouse models of PD. Therefore, the aim of this work is to determine the effect of pramipexole on T regulatory cells of human origin and its possible neuroprotective effect on hESC‐derived dopaminergic neurons.

## 2 MATERIALS AND METHODS

### 2.1 Purification of regulatory T cells populations

CD4+ cells were purified from buffy coats taken from healthy subjects (nine buffy coats; 5 male and 4 female donors) using the Rosette Human CD4+ T Cell Enrichment Cocktail (StemCell Technologies, Vancouver, Canada). The cells were then washed twice with 2% fetal bovine serum (FBS) in phosphate‐buffered saline (PBS). To purify Treg cells by flow cytometry, CD4+ cells were labeled with the following antibodies: CD4 allophycocyanin‐cyanine 7 (APC‐Cy7) (BD Bioscience, IgG1k isotype), CD25 allophycocyanin (APC) (BD Bioscience, IgG1k isotype), and CD127 phycoerythrin‐cyanine 7 (PE‐Cy7) (BD Bioscience, IgG1k isotype). The cells were incubated for 30 min at 4°C, protected from light. The cells were then passed through a 40‐micron filter and the volume was adjusted to 10^7^ cells/mL using fluorescence‐activated cell sorting (FACS) solution. The cells were purified on a BD FACSAria I cytometer to a purity greater than 96%, as shown in Figure S1.

### 2.2 Enrichment in IL‐10+ CD4+ cells

CD4+ T cells were purified from buffy coats taken from healthy subjects (five buffy coats; 3 male and 2 female donors) using the RosetteSep human CD4+ T cell enrichment cocktail (Stemcell Technologies), following the manufacturer's instructions. The cells were stimulated with anti‐CD3/CD28 ImmunoCult™ Human CD3/CD28 T Cell Activator (StemCell Technologies) for 4 days, and then treated for 24 h with pramipexole at concentrations of 2 and 200 ng/mL. Responder cells were labeled and isolated according to IL‐10 secretion using the IL‐10 secretion assay Cell Enrichment and Detection Kit (Miltenyi Biotec, Bergisch Gladbach, Germany). Briefly, stimulated cells were labeled with IL‐10 specific capture reagent and incubated briefly at 37°C. The cells were then labeled with a second IL‐10‐specific antibody conjugated to R‐phycoerythrin (PE) for detection by flow cytometry. IL‐10 secreting cells can be magnetically labeled with anti‐PE MicroBeads and enriched on a MACS® Column under the magnetic field of a MACS separator. Magnetically labeled cells are retained on the MACS column, whereas unlabeled cells pass through. The retained cells can then be eluted as a positively selected cell fraction enriched for cytokine‐secreting cells.

### 2.3 Differentiation to dopaminergic neurons

To investigate the effect of stimulating immunoregulatory populations with dopaminergic agonists on neuroprotection, the cells were placed in direct contact with human dopaminergic neurons via co‐culture. Human H9 (WA09) embryonic stem cells (WiCell, Madison, WI, USA), previously transfected with a lentiviral vector to express green fluorescent protein (GFP),^19^ were used. H9‐GFP cells were seeded on Matrigel (Corning) and allowed to proliferate up to 70% using mTeSR™1 medium. Differentiation into dopaminergic neurons was then initiated by dual inhibition of the suppressor of mothers against decapentaplegic (SMAD) and floor plate induction, following a previously described protocol.^10^ Briefly, the cells were maintained in knockout serum replacement medium (KSR) containing Knockout DMEM (Gibco, USA), 20% serum replacement (Gibco, USA), 1× non‐essential amino acids (Gibco, USA), 1 mM GlutaMAX (Gibco, USA), 0.1 mM 2‐mercaptoethanol (Gibco, USA), 0.5% penicillin/streptomycin (Gibco, USA), and 8 ng/mL fibroblast growth factor (bFGF) (Invitrogen, USA). Using KSR as a base, the cultures were exposed to LDN193189 (100 nM, Stemgent; days 0–11), SB431542 (10 μM, Tocris; days 0–5), SAG (1 μM, Sigma; days 1–9), Purmorphamine (2 μM, Stemgent; days 1–9), FGF8 (0. 1 μg/mL, Prepotech; days 1–7), and CHIR99021 (3 μM, Sigma Aldrich, Germany; days 3–13). The KSR medium was gradually changed to B27‐supplemented neurobasal medium (NB/B27; Invitrogen, USA) from day 5 to day 10. Starting on day 11, the culture was maintained in neurobasal B27 maturation medium supplemented with brain‐derived neurotrophic factor (BDNF) (20 ng/mL, Prepotech), ascorbic acid (0.2 μM, Sigma), glial cell line‐derived neurotrophic factor (GDNF) (20 ng/mL, Prepotech), transforming growth factor beta‐3 (TGFβ‐3) (1 ng/mL, Peprotech, USA), dibutyryl cAMP (0.5 μM; Sigma Aldrich, USA), and DAPT (10 μM; Sigma Aldrich, USA). On day 21, the cells were dissociated using TrypLE Express (Life Technologies) and seeded onto 96‐well flat‐bottom plates (50,000 cells/well). Thereafter, the cells were maintained in maturation medium until the end of the experiments. All cultures were maintained at 37°C under 5% CO_2_.

### 2.4 Co‐culture

To assess the neuroprotective effect of Tregs treated with the dopaminergic agonist, these cells were purified by flow cytometry as described previously. To study the effect of activation, Treg cells were activated with anti‐CD3/CD28 ImmunoCult™ Human CD3/CD28 T Cell Activator (StemCells Technology, Canada). Tregs were then sorted into two groups: group 1 was pre‐activated with anti‐CD3/CD28 (5 μg/mL) for 4 days, and the second group received no activation. The regulatory cells were then plated in a 96‐well U‐bottom plate and incubated for 24 h with the dopaminergic agonist pramipexole at concentrations of 2 or 200 ng/mL. After incubation, the cells were centrifuged at 560 × *g* for 15 min and the supernatant was removed; the cells were resuspended in maturation medium, immediately plated onto wells containing mature dopaminergic neurons on differentiation day 28–32, and allowed to interact for 24 h. The supernatant was then removed and stored at −80°C until use.

### 2.5 Neurite growth assays

To observe whether supernatants from the cocultures with IL‐10+ Tregs had any effect on neurite outgrowth, 10^5^ differentiated dopaminergic neurons derived from human H9 (WA09) embryonic stem cells were placed in a 96‐well flat‐bottom plate and then stimulated for 48 h with 100 μL of supernatants from the cocultures of IL‐10‐producing regulatory T cells with neurons, plus 100 μL of maturation medium. Neurons were then washed with PBS at room temperature and fixed with 4% p‐formaldehyde for 20 min. They were then subjected to immunostaining.

### 2.6 6‐OHDA induced damage model

6‐Hydroxydopamine (6‐OHDA) has been widely used in models of neuronal injury. A stock solution of 10 mM 6‐OHDA (Sigma Aldrich, Germany) was prepared just prior to the experiment in 200 μM ascorbic acid. The stock was then diluted in maturation medium to the desired concentration (6‐OHDA, 50 μM). A 6‐OHDA pulse was applied for 4 h as previously described.^20^, ^21^ After 4 h, 6‐OHDA solution was replaced with fresh maturation medium. Twenty hours after 6‐OHDA treatment, control and 6‐OHDA‐treated cells were washed with PBS, fixed with p‐formaldehyde, and processed for immunohistochemical assays.

### 2.7 Immunohistochemistry

The cells were blocked and permeabilized for 1 h using PBS containing 0.3% Triton X 100 and 10% normal goat serum. The cultures were then treated with the following primary antibodies, rabbit anti‐tyrosine hydroxylase (TH) 1:1000 (P40101, Labome, USA) and mouse anti‐beta‐3 tubulin (TUJ1) 1:3000 (821202, CiteAb, UK) in PBS and 10% normal goat serum, at 4°C, for 12 h. Anti‐rabbit‐Alexa Fluor 568 and anti‐mouse‐Alexa Fluor 647 were used as secondary antibodies (1:1000). This immunohistochemistry protocol was used for both survival analysis and neurite outgrowth assays.

### 2.8 Image analysis

Five images per well were captured using an epifluorescence microscope (Cytation 5, Biotek, USA). The percentage of area positive for the markers of interest (TUJ1+ and TH+) was quantified using the ImageJ software (USA, NIH). The percentage of dopaminergic neurons was calculated by taking the signal for TUJ1+ (neuronal population) as 100% and TH+ as the signal for dopaminergic neurons.

To observe neuronal growth, 15 photographs per well were taken under an epifluorescence microscope (Cytation 5, Biotek, USA); the length of primary TH‐positive neurites was measured as reported by Pemberton et al. (2018).^22^ Sixty‐five measurements were made per condition. Images were processed using the NeuronJ plugin in Fiji software (ImageJ) v.1.54f. (Rasband, W.S., ImageJ, U.S. National Institutes of Health, Bethesda, Maryland, USA, https://imagej.nih.gov/ij/, 1997–2018).

### 2.9 RNA extraction, cDNA synthesis, and pre‐amplification

Total ribonucleic acid (RNA) was extracted using the RNeasy mini kit (Qiagen, Germany) through columns, following the kit's instructions. RNA was quantified by absorbance (260/280) in a Synergy kit using the Take 3 microvolume plate (Biotek, USA).

Total RNA (50 ng of total RNA in a 20 μL reaction volume) was used to synthesize complementary DNA (cDNA) using the OneStep RT PCR kit (Qiagen, Germany). A mixture of 96 primer pairs (2 μM) was used, and the solution was incubated under the following conditions: 50°C for 30 min, 95°C for 15 min, followed by 18 cycles of pre‐polymerase chain reaction (PCR) amplification at 94°C for 30 s, 60°C for 60 s, and 72°C for 60 s. Pre‐amplification products were diluted 20‐fold with Tris/ethylenediaminetetraacetic acid (TE) buffer.

### 2.10 Real‐time PCR

To assess gene expression, 61 genes were selected according to their importance in immunoregulatory and neuroprotective roles, with different expression levels in the immunoregulatory populations studied. Eight reference genes were included for relative expression analysis. Primers were designed using a Deltagene assay (Fluidigm, South San Francisco, USA) and are listed in Table S1.

qPCR was performed on a BioMark 96.96 Integrated Fluidic Circuit (IFC) (Fluidigm, South San Francisco, CA, USA), combining 96 primer pairs with 96 pre‐amplified samples for multi‐parallel qPCR reactions. The sample mix included SSoFast Master Mix (Bio‐Rad, Hercules, CA, USA) and 20× Sample Loading Reagent (Fluidigm, South San Francisco, CA, USA) for each pre‐amplified cDNA. Each of the 96 assay mixtures was prepared with 100 μM of its respective primer pair and DNA Suspension Buffer (Buffer TE). Thermal cycling and detection were performed on a BioMark instrument (Fluidigm, South San Francisco, CA, USA) under the following conditions: 95°C for 3 min, 25 cycles at 95°C for 30 s, 62°C for 20 s. Melt curve analysis (65–95°C) was performed after PCR amplification to measure each amplicon. Cq values and amplification curves were analyzed using Standard Bio Tools Real‐Time PCR Analysis 1.0.1 software (Standard Bio Tools Inc., USA).

### 2.11 Gene expression data analysis

The Qbase+ software (BioGazelle, Zwijnaarde, Belgium) was used to determine the expression stability of the reference genes using the geNorm and geNorm V algorithms,^23^ according to the Minimum Information for Publication of Quantitative Real‐Time PCR Experiments (MIQE) guidelines^23^, ^24^ (Figure S2). The relative expression of each gene was determined using two independent donor samples (biological replicates) and three technical replicates per sample, and it was compared with the selected reference genes. Raw qPCR data (cycle quantification [Cq] values) were normalized to the geometric mean of the most stable reference genes for the cell population. SDHA and UBE2D2 were the most stable for Tregs activated with anti‐CD3/CD28. The relative expression of each gene in cells treated with pramipexole at a dose of 2 or 200 ng/mL was calculated, compared with unstimulated cells, and a fold‐change matrix was constructed. This matrix was used for Z‐transformation and an analysis of differentially expressed genes (DEGs).

Heatmapping was performed by transforming RT‐qPCR data into *Z*‐scores and clustering them using an average linkage method with a Pearson correlation. This was done on the Heatmapper website (www.heatmapper.ca/expression). The *Z*‐scores were then used to calculate a *Z*‐ratio, where a *Z*‐ratio >1.0 or <−1.0 was considered significant.

Path analysis was performed based on the clustering of genes derived from the heatmaps. This analysis was performed in the gene enrichment tool Enrichr using the Reactome 2022 gene set library (https://maayanlab.cloud/Enrichr/).^25^

### 2.12 Statistical analysis

All values are expressed as mean ± standard deviation (SD). The distribution of the results was assessed by the Shapiro–Wilk test.

To assess the neuroprotective effect of regulatory cells, the differences in the percentage of TH+/TUJ1+ neurons were analyzed by one‐way ANOVA, followed by Dunnett's post‐hoc test for multiple comparisons. To determine the difference in the length of primary neurites, data were analyzed using a one‐way ANOVA, followed by Dunnett's post‐hoc test.

All statistical analyses were performed using the Graphpad Prism 9.5.0 software (GraphPad Software Co., California, USA).

## 3 RESULTS

### 3.1 Effect of pramipexole treatment on Tregs in a 6‐OHDA‐induced neuronal damage model

**FIGURE 1:**
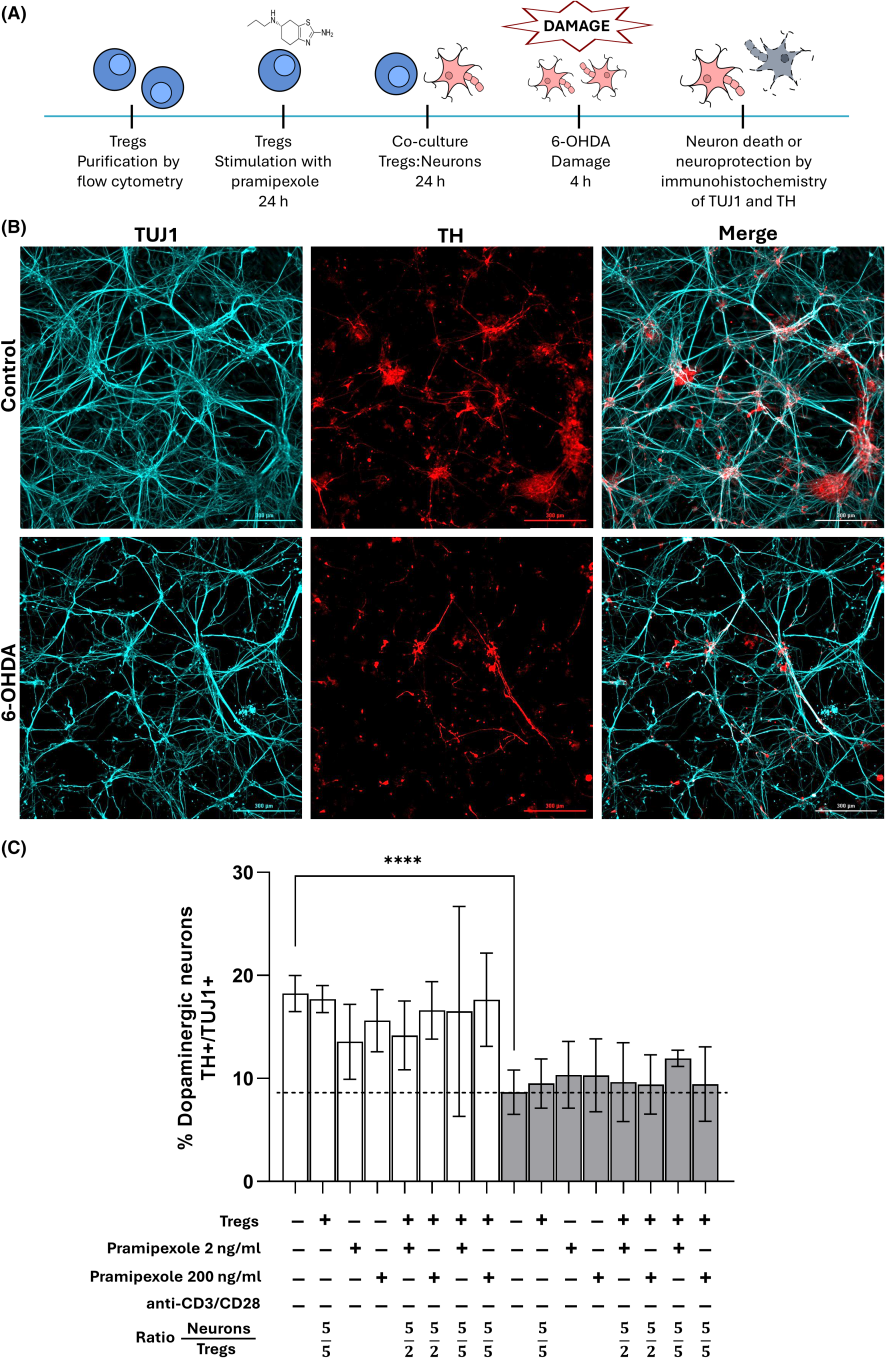
In vitro model to evaluate the neuroprotective effect of non‐activated Treg cells. (A) Schematic representation of the in vitro model to evaluate the neuroprotective effect of non‐activated Treg cells on neurons. (B) Cell culture of human dopaminergic neurons. Representative confocal microscopy images of dopaminergic neurons. Cells were treated with 50 μM 6‐OHDA for 4 h. The markers TUJ1 (cyan) and TH (red) are shown. Scale bar: 300 μm. (C) Decrease in the proportion of dopaminergic (TH+) neurons after incubation with 6‐OHDA and the effect of coculture with pramipexole‐treated Treg cells. ****p < 0.0001, one‐way ANOVA and Dunnett's post hoc test were used to compare 6‐OHDA‐treated, control neurons (dotted line) with the other conditions. Bars in all graphs indicate the mean percentage ± SD of at least three independent experiments. White bars indicate control neurons; gray bars indicate 6‐OHDA‐damaged neurons.

H9 human embryonic stem cells expressing GFP were induced to differentiate into mesencephalic dopaminergic neurons. After 28 days, the differentiated cells expressed the neuronal markers beta 3‐tubulin (identified by the TUJ1 antibody) and tyrosine hydroxylase (identified by the TH antibody) as dopaminergic markers. 6‐OHDA was administered to develop a model of dopaminergic neuron damage, and concentration‐response curves were plotted to determine the sensitivity of cultured dopaminergic neurons to this drug. As a result, 50 μM 6‐OHDA was chosen for all experiments. To test whether Treg cells stimulated with the dopamine receptor agonist pramipexole (at a dose of 2 or 200 ng/mL) could protect dopaminergic neurons from 6‐OHDA‐induced damage, they were co‐cultured with neurons (Figure 1A). As shown in Figure 1B, a significant decrease in the percentage of TH+/TUJ1+ surviving neurons was observed in 6‐OHDA‐treated neurons compared with control cells. When co‐cultured with pramipexole‐treated Tregs, 6‐OHDA still caused a decrease in the percentage of TH+/TUJ1+ neurons (Figure 1C).

**FIGURE 2:**
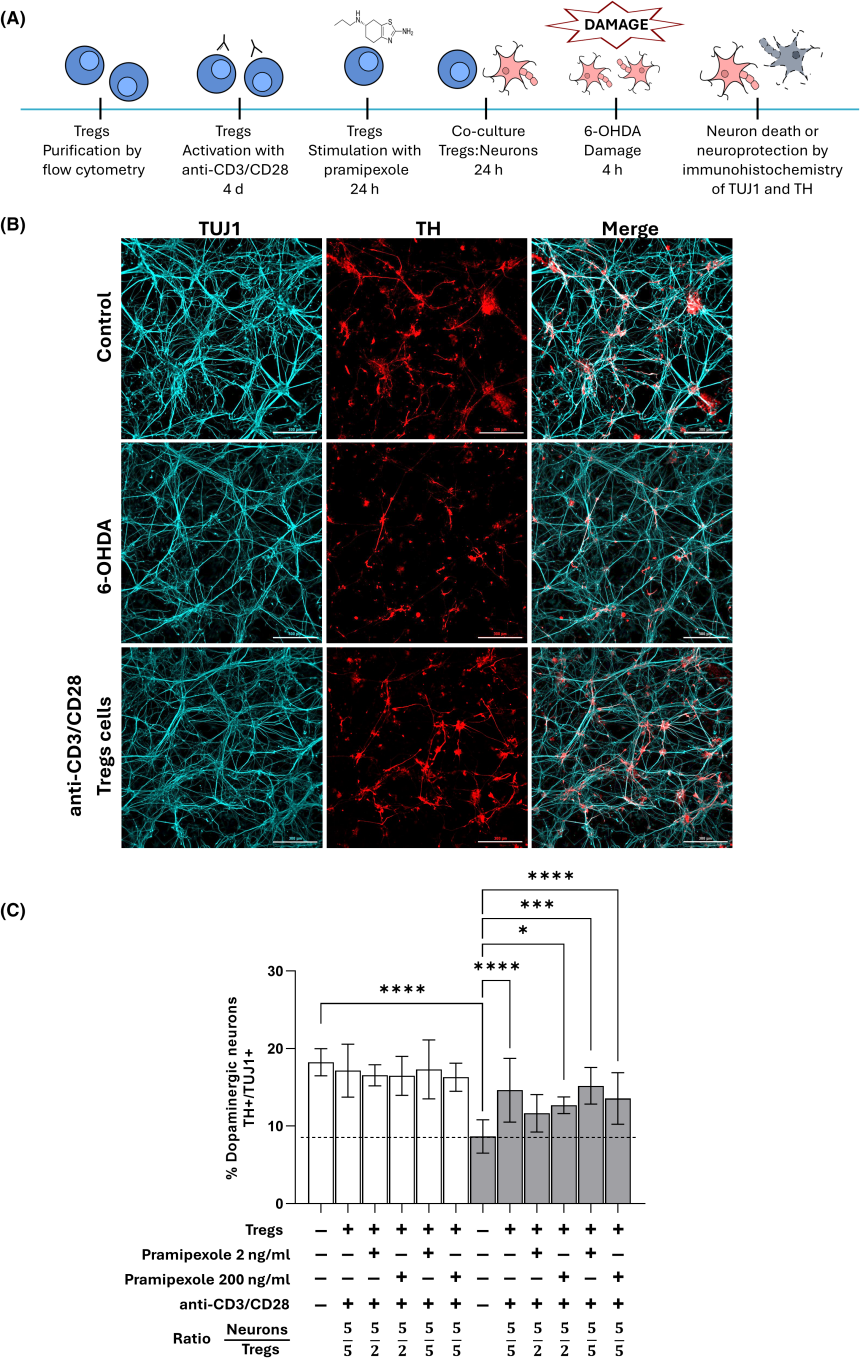
Activated Treg cells protected dopaminergic neurons against 6‐OHDA toxicity independently of pramipexole treatment. (A) Schematic representation of the in vitro model to evaluate the neuroprotective effect of CD3/CD28‐activated Treg cells on neurons. (B) Cell culture of human dopaminergic neurons. Representative confocal microscopy images of dopaminergic neurons. Cells were treated with 50 μM 6‐OHDA for 4 h. The markers TUJ1 (cyan) and TH (red) are shown. Scale bar: 300 μm. (C) Effect of co‐culturing with Treg cells preactivated with anti‐CD3/CD28 and treated with pramipexole on the percentage of dopaminergic neurons after 6‐OHDA‐induced damage. *p < 0.05; ***p < 0.001; ****p < 0.0001, one‐way ANOVA and Dunnett's post hoc test were used to compare 6‐OHDA‐treated, control neurons (dotted line) with the other conditions. Bars in all graphs indicate the mean percentage and SD of at least three independent experiments. White bars indicate control neurons; gray bars indicate 6‐OHDA‐damaged neurons.

An inflammatory phenomenon at the CNS level is observed in PD, leading to an overactivation of the immune response. Therefore, Treg cells were preactivated with anti‐CD3/CD28 for 4 days (Figure 2A). A significant increase in the percentage of dopaminergic neurons with respect to damaged neurons was observed when preactivated Tregs were co‐cultured with neurons in ratios of 5:5 and 5:2 and treated with 200 ng/mL of pramipexole, and in a 5:5 ratio when treated with 2 ng/mL of pramipexole, as shown in Figure 2B,C, respectively. In addition, a significant increase in the percentage of dopaminergic neurons was observed when Treg cells not treated with pramipexole were co‐cultured with neurons in a 5:5 ratio.

### 3.2 Effect of pramipexole treatment on different proportions of regulatory T cells in a model of 6‐OHDA‐induced damage

**FIGURE 3:**
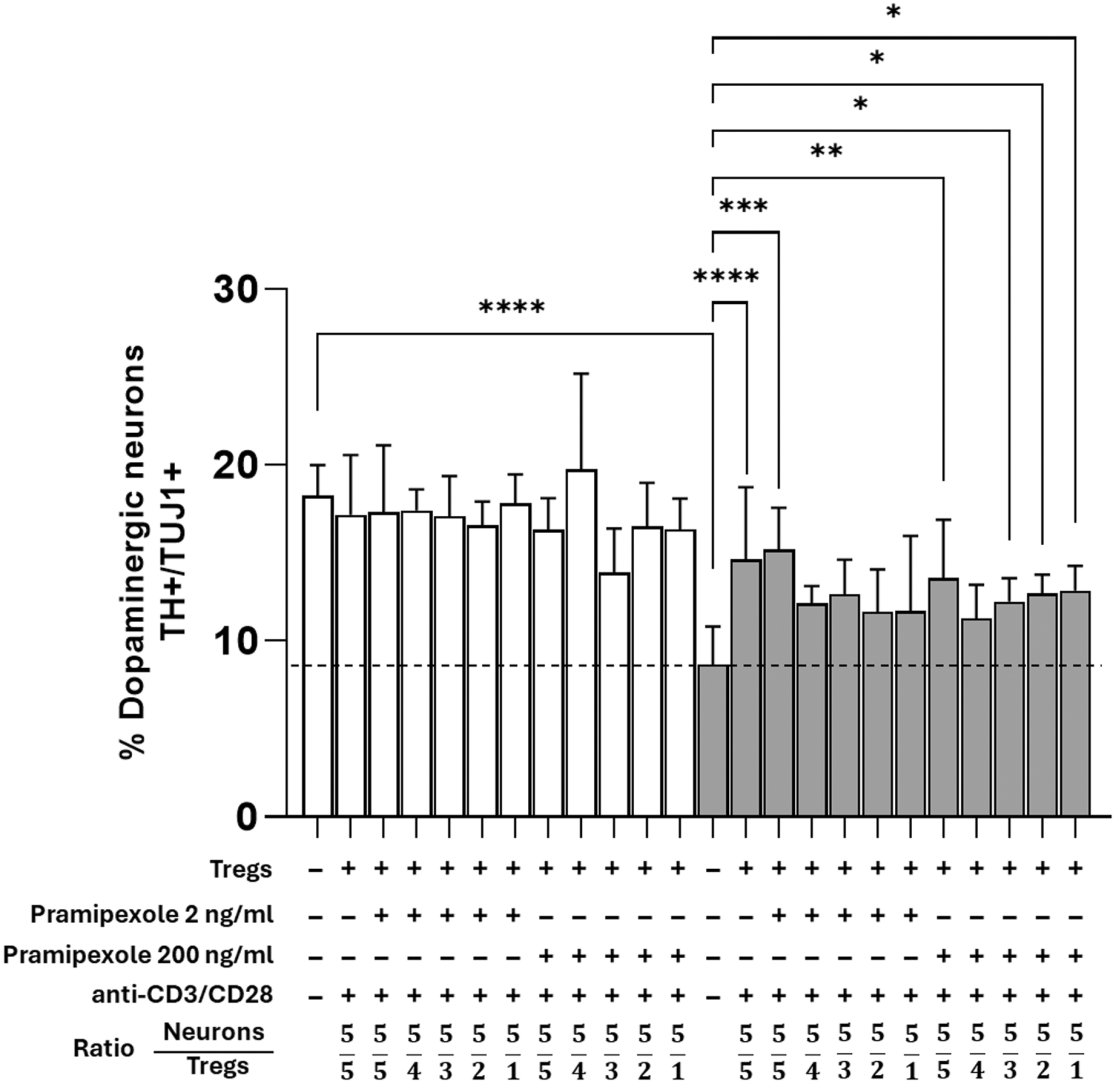
The protective effect of activated Tregs is preserved at different neuron:Treg ratios. Effect on the percentage of dopaminergic neurons of co‐cultures with different proportions of anti‐CD3/CD28‐activated, pramipexole‐treated Treg cells after 6‐OHDA damage. *p < 0.05; **p < 0.01; ***p < 0.001; ****p < 0.0001, one‐way ANOVA and Dunnett's post hoc test were used to compare 6‐OHDA‐treated, control neurons (dotted line) with the other conditions. Bars in all graphs indicate the mean percentage ± SD of at least three independent experiments. White bars indicate control neurons; gray bars indicate 6‐OHDA‐damaged neurons.

To investigate whether different ratios of regulatory cells might affect their ability to protect neurons from damage by 6‐OHDA, regulatory T cells activated with anti‐CD3/CD28 and treated with pramipexole were co‐cultured at neuron:Treg ratios of 5:5, 5:4, 5:3, 5:2, and 5:1, as shown in Figure 3. Among the Tregs treated with 2 ng/mL pramipexole, only those cells co‐cultured in a 5:5 ratio significantly increased the percentage of dopaminergic neurons with respect to damaged 6‐OHDA cells. Meanwhile, for cells treated with 200 ng/mL pramipexole, all neuron:Treg ratios except for the 5:4 ratio caused a significant increase in the percentage of dopaminergic neurons with respect to 6‐OHDA‐damaged cells. In contrast, as shown in Figure S3, when Treg cells were not activated with anti‐CD3/CD28 prior to co‐culture with neurons, they failed to increase the percentage of dopaminergic neurons with respect to damaged neurons at all neuron:Treg ratios.

### 3.3 Effect of pramipexole treatment on IL‐10‐producing regulatory T cells in a 6‐OHDA‐induced damage model

**FIGURE 4:**
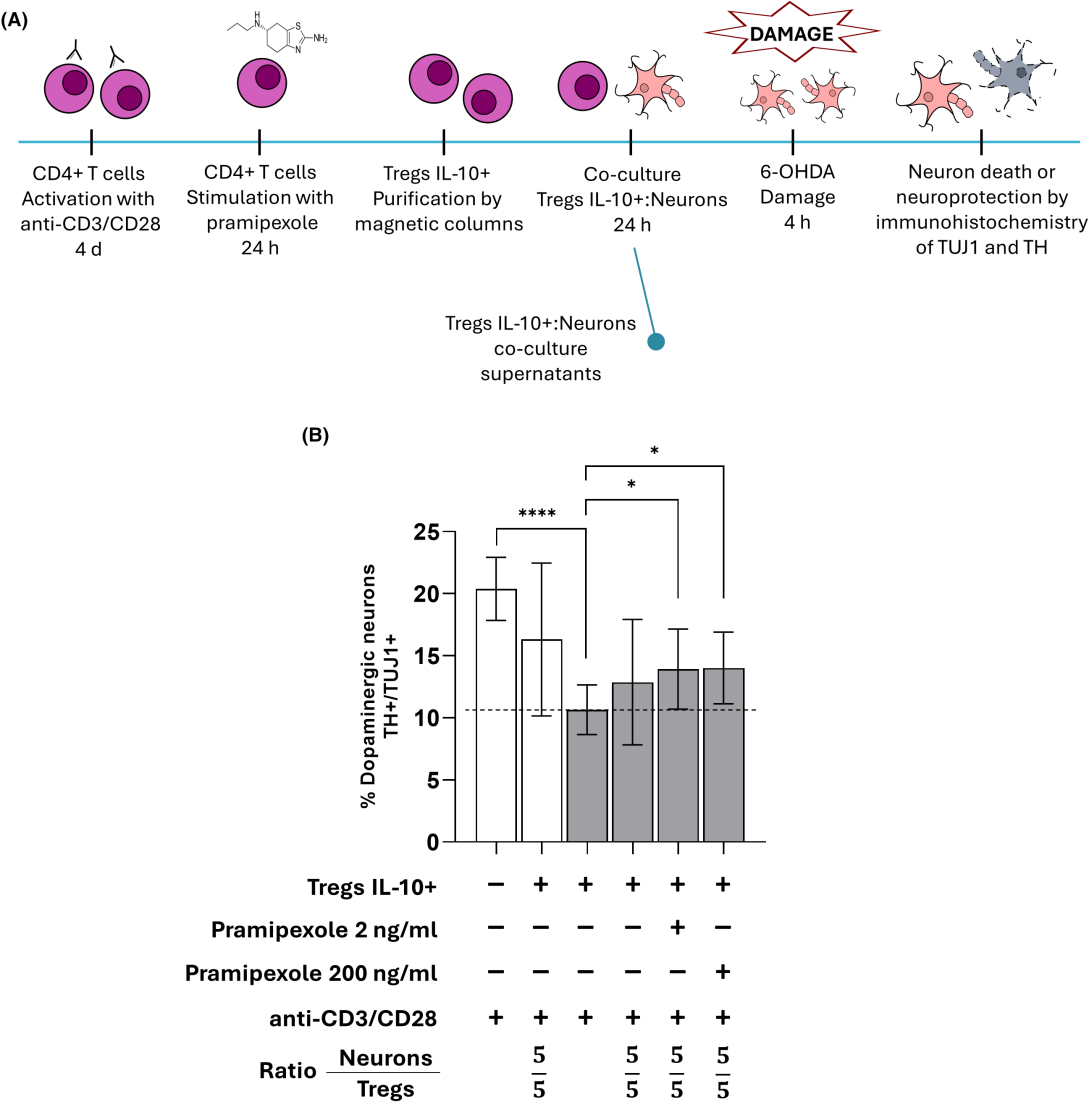
CD3/CD28‐activated, pramipexole‐treated IL‐10+ Tregs cells protect dopaminergic neurons against 6‐OHDA‐induced damage. (A) Schematic representation of the in vitro model to evaluate the neuroprotective effect of IL‐10+ Tregs. (B) Purified IL‐10+ Tregs protect dopaminergic neurons against 6‐OHDA toxicity. Effect on the percentage of TH+/TUJ1+ dopaminergic neurons of co‐culture with IL‐10+ Tregs preactivated with anti‐CD3/CD28 and treated with pramipexole after 6‐OHDA damage. *p < 0.05; ****p < 0.0001, one‐way ANOVA and Dunnett's post hoc test were used to compare 6‐OHDA‐treated, control neurons (dotted line) with the other conditions. Bars in all graphs indicate the mean percentage ± SD of at least three independent experiments. White bars indicate control neurons; gray bars indicate 6‐OHDA‐treated neurons.

To investigate whether the anti‐inflammatory cytokine IL‐10 is involved in the neuroprotective effect of Tregs, CD4+ T cells were activated with anti‐CD3/CD28, treated with pramipexole at a concentration of 2 or 200 ng/mL, and then enriched for IL‐10‐producing Tregs. These cells were co‐cultured at a 5:5 neuron:Treg ratio (Figure 4A). A significant increase in the percentage of dopaminergic neurons with respect to damaged neurons was observed when Tregs were stimulated with 2 and 200 ng/mL pramipexole, as shown in Figure 4B.

### 3.4 Effect of IL‐10+ Treg supernatants on neurite outgrowth

**FIGURE 5:**
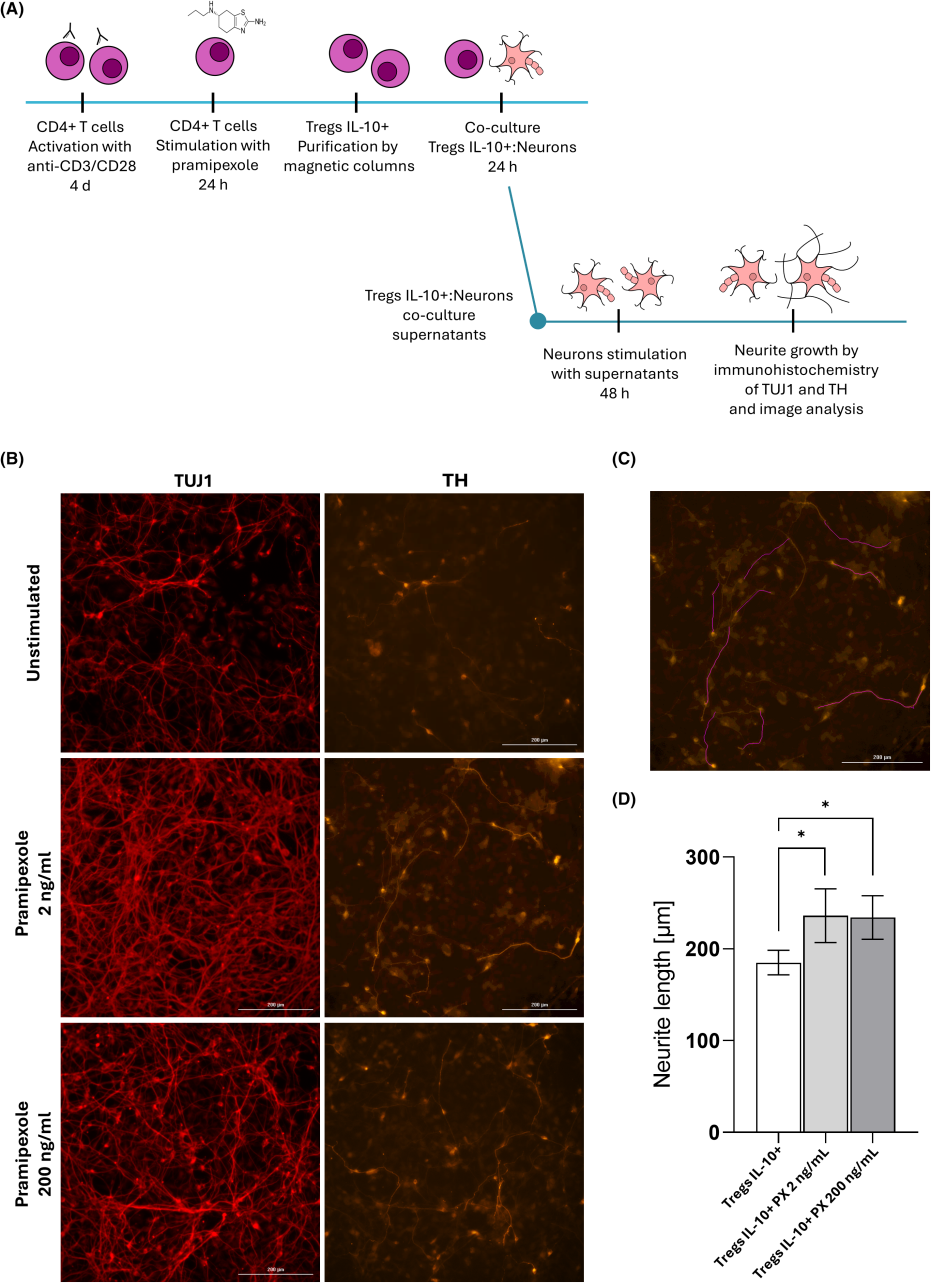
Supernatants from IL‐10+ Tregs cultures increase primary neurite length in dopaminergic neurons not challenged with 6‐OHDA. (A) Schematic representation of the in vitro model to assess the effect of supernatants derived from co‐cultured IL‐10+ Tregs and neurons on neurite outgrowth in dopaminergic neurons. (B) Representative fluorescence image of reactivity to the dopaminergic neuron markers TH (orange) and TUJ1 (red). Scale bars: 200 μm. (C) Example of primary neurite length measurement using the software Fiji and the NeuronJ plugin. (D) Primary neurite length after 48‐h stimulation with supernatants of IL‐10+ Tregs co‐cultured with undamaged dopaminergic neurons. *p < 0.05, one‐way ANOVA and Dunnett's post hoc test were used to compare control and pramipexole‐treated cells. Bars in the graph indicate the mean percentage ± SD of at least three independent experiments.

To investigate whether the products released by pramipexole‐treated IL‐10+ Tregs have an effect on the length of neurites, supernatants from co‐cultures were brought into contact with fresh neurons (Figure 5A). As shown in Figure 5B, a significant increase in the length of primary neurites of dopaminergic neurons was observed upon contact with supernatants of IL‐10+ Tregs treated with 2 or 200 ng/mL of pramipexole and co‐cultured with undamaged dopaminergic neurons.

### 3.5 Effect of pramipexole treatment on the expression of genes related to anti‐inflammation and neuroprotection in regulatory T cells

**FIGURE 6:**
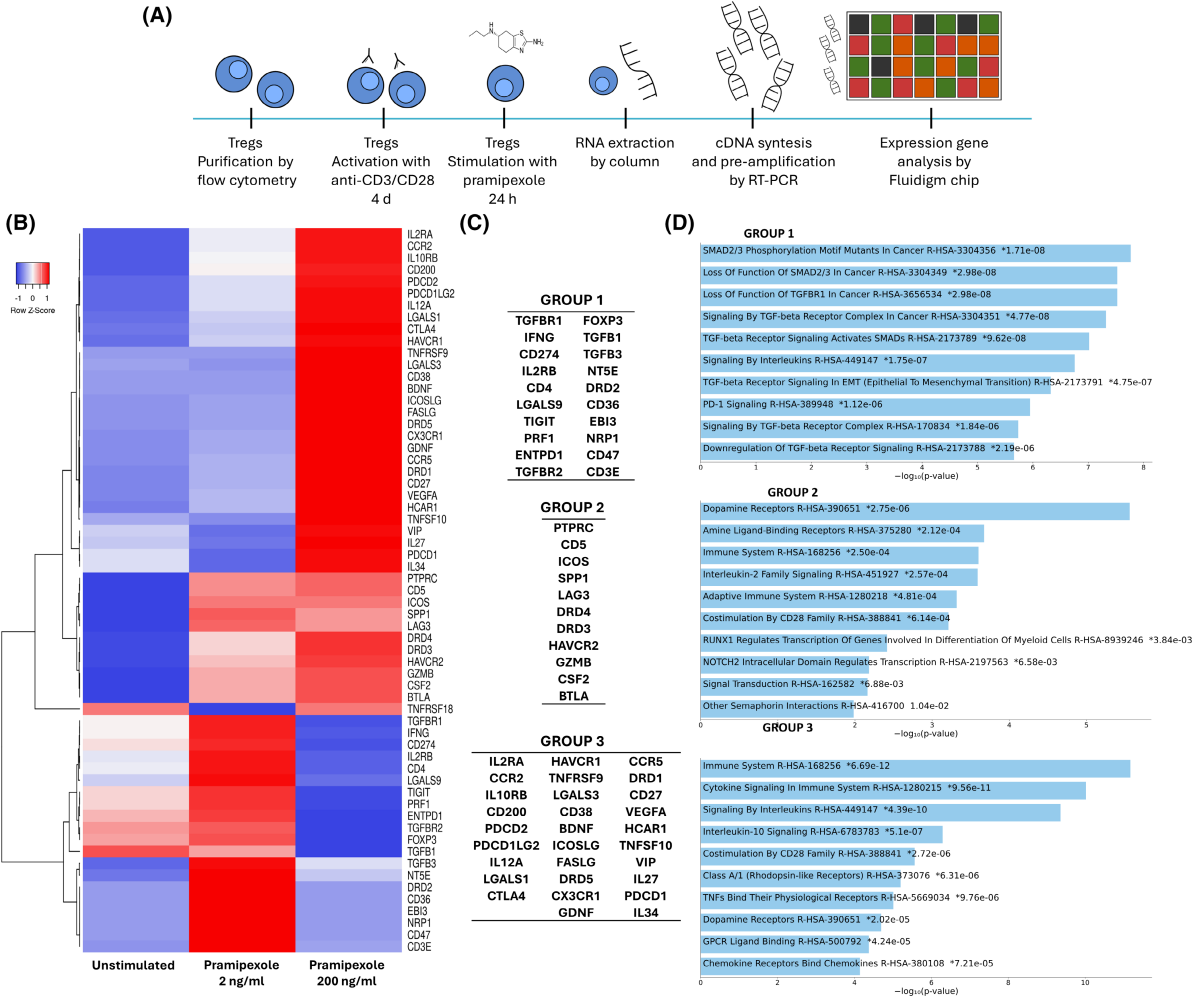
Pramipexole‐induced gene expression changes in activated Treg cells. (A) Schematic representation of the in vitro model used to assess the effect of pramipexole treatment on gene expression in Tregs (B) Heatmap showing hierarchical clustering of gene expression in anti‐CD3/CD28‐activated Treg cells stimulated with pramipexole at concentrations of 2 and 200 ng/mL, with respect to unstimulated cells. Data are reported as a *Z*‐score, indicating the change in expression for each concentration of pramipexole relative to the average change in the unstimulated condition. Negative *Z*‐score values indicate a decrease (blue), and positive values indicate an increase (red) in the expression of each gene. (C) Clustering of upregulated genes. (D) Enrichment path analysis of DEGs using the Reactome 2022 set library, and the corresponding *p*‐values. The top 10 enriched terms for the input gene set are shown based on the −log_10_(*p*‐value), with the actual *p*‐value shown next. Colored bars correspond to terms with significant *p*‐values (<0.05). An asterisk (*) next to a *p*‐value indicates that the term also has a significant adjusted *p*‐value (<0.05).

Treg cells have been shown to protect neurons against 6‐OHDA‐induced damage. Therefore, to investigate which molecules in Treg cells are affected by pramipexole treatment, purified Treg cells were activated with anti‐CD3/CD28 and treated with pramipexole at concentrations of 2 or 200 ng/mL. Total RNA from the cells was used to measure the expression of 61 genes (Table S1) related to regulation and neuroprotection (Figure 6A). Gene expression in regulatory T cells is shown as a heat map in Figure 6B. Genes were stratified into three groups according to their expression level. Group 1 showed higher expression when treated with 2 ng/mL pramipexole compared with unstimulated cells. Group 2 showed higher expression levels with both doses of pramipexole. Group 3 showed higher expression with 200 ng/mL pramipexole (Figure 6C).

It should be noted that pramipexole at a dose of 200 ng/mL caused overexpression of D1‐like dopamine receptor genes, whereas both doses of pramipexole upregulated D2‐like receptor genes, DRD3 and DRD4. Notably, DRD2 receptor genes were only upregulated at a dose of 2 ng/mL.

At the same time, genes related to neuroprotection were upregulated. In Group 1, TGFB1, TGFB3, CD47, ENTPD1 (CD39), and NRP1 were activated; in Group 2, the genes PTPRC (CD45), SPP1 (osteopontin), and CSF2 (GM‐CSF) were activated; and in Group 3, the genes BDNF, VEGFA, CD200, VIP, GDNF, LGALS1 (galectin 1), and HCAR1 were upregulated.

To determine the biological pathways or processes in which the genes of each group are involved, the Reactome database was used. The results are shown in Figure 6D. Group 1 genes were found to be mainly associated with the TGFβ modulation pathway, Group 2 genes were found to be associated with the dopamine receptor pathway, and Group 3 genes were found to be associated with the immune system and cytokine signaling.

## 4 DISCUSSION

Parkinson's disease is characterized by the death of dopaminergic neurons in the *SNpc* due to the presence of intracellular protein inclusions called Lewy bodies, mainly formed by α‐Syn. This has been attributed to dysfunction in proteostasis, genetic factors predisposing to errors in post‐translational modifications, or failures of clearance systems, such as the ubiquitin‐proteasome or phagolysosomal systems. All of these factors promote neuronal death and induce a persistent state of neuroinflammation.^26^, ^27^

Regulatory T cells have been proposed to exert neuroprotective effects in murine and in vitro models.^18^, ^28^, ^29^ We now know that regulatory cells express receptors for dopamine, and thus they can be affected by dopaminergic treatment.^30^ At the same time, pramipexole itself has been proposed to have a direct neuroprotective effect.^31^, ^32^ In this study, we analyzed the effect of treatment on regulatory cells (activated and non‐activated) in their ability to protect 6‐OHDA‐treated neurons from damage.

Our results showed that pramipexole alone did not protect neurons from damage. This contrasts with previous reports in mouse models, where low concentrations of pramipexole protect from MPTP‐induced damage by reducing the amount of toxic compound bound to dopamine receptors.^31^ It has also been reported that trans‐dermally administered pramipexole protects against from MPTP‐induced damage, and restored motor skills damaged by the stimulus. In addition, pramipexole has been observed to reduce the amount of ROS and the activation of caspase‐3 and ‐9 pathways, and to increase dopamine production.^32^ A possible explanation for this lack of activity could be in the timing of pramipexole administration, as in animal models pramipexole is administered after injury. Our study focuses on a preventive effect of pramipexole‐treated Tregs, and whether the treatment could contribute to preventing the death of undamaged dopaminergic neurons during disease progression. This idea is supported by the fact that we observed that supernatants from pramipexole‐treated IL‐10+ cells significantly increased the length of primary neurites at both drug concentrations, which could indicate an improvement in neuronal function.^33^

On the other hand, Treg cells were shown to be neuroprotective only after activation with anti‐CD3/CD28. It has been reported that stimulation of Tregs with anti‐CD3/CD28, rapamycin, or retinoic acid increases their proliferation and enhances their regulatory capacity, which could improve their function in the treatment of autoimmune diseases or transplant rejection.^34^ This suggests that activation of the suppressive immune response is required to promote a neuroprotective effect of regulatory T cells.

It is noteworthy that this neuroprotective effect of Tregs is independent of pramipexole treatment, as well as of the Treg:neuron ratio. The latter fact suggests that the neuroprotective effect of Tregs may be mediated by a soluble factor and not be due to cell–cell contact, as it has been observed in mouse models.^18^ Thus, our results suggest that IL‐10‐producing Tregs have a neuroprotective effect, which reinforces the role of IL‐10, a cytokine with anti‐inflammatory activity; this effect would be mediated by the IL‐10 receptor through the JAK/STAT3 pathway, leading to decreased cleavage of caspase‐3 and ‐9, thus reducing neuronal death in models of PD based on LPS‐induced damage.^35^ This is consistent with reports on murine models of PD, where Treg protection against damage is attributed not only to cell–cell contact but also to contact‐independent mechanisms, such as cytokine production.^18^ However, the effect of IL‐10 alone against 6‐OHDA‐induced damage should be addressed, as well as the concentration at which it is produced, as it has also been reported that a sustained IL‐10 production may have the opposite effect and cause inflammation in the CNS.^36^

On the other hand, mRNA analysis of Tregs after treatment with pramipexole at concentrations of 2 and 200 ng/mL showed a differential effect on the expression of genes related to suppressor activity (Figure 6B). In addition, a change in the expression of genes encoding D1‐like and D2‐like dopamine receptors was observed. In particular, both DRD3 and DRD4 genes showed increased expression levels after treatment with 2 and 200 ng/mL pramipexole. It should be noted that the increased expression of the DRD2 receptor was only observed at a pramipexole concentration of 2 ng/mL. On the other hand, D1‐like receptors increased their expression only at a concentration of 200 ng/mL. This is relevant because it has been reported that activation by D1‐like dopamine receptors suppresses the regulatory capacity of CD4+ and CD8+ Tregs.^16^, ^37^, ^38^, ^39^ Interestingly, specific activation of the D‐5 receptor enhances the regulatory capacity of these cells.^40^ In addition, specific activation of D‐3 and D‐1/D‐5 in human T cells has been shown to promote TNFα production, whereas activation of D‐2 and D1/D‐5 promotes IL‐10 production.^41^ There is evidence that pramipexole induces an increase in DR2 expression in both animal models and clinical trials, indicating that this dopaminergic agonist improves motor symptoms in PD and that its mechanism of action may be related to its ability to increase the expression of the D2 receptor.^42^, ^43^

Remarkably, specific activation of the D‐2 receptor in Tregs in a mouse model of PD enhances the neuroprotective capacity of the cells against MPTP‐induced damage.^44^

Interestingly, genes from Groups 1 and 3 are related to neuroprotection Genes in Group 1 (which showed a higher expression when treated with 2 ng/mL pramipexole) include CD47, NRP1, TGFB1, and TGFB3, all of which were overexpressed. CD47 has been reported to activate the SHP‐1 and ‐2 pathways upon binding to SIRPα, thereby regulating neuronal survival and inhibiting apoptosis.^45^ On the other hand, Group 3 genes (which showed a higher expression after treatment with 200 ng/mL pramipexole) include neuroprotective genes such as VEGFA, VIP, Galectin 1 (LGALS1), HCAR1, BDNF, and GDNF.^46^, ^47^, ^48^, ^49^, ^50^ In particular, BDNF and GDNF are neurotrophic factors that have been shown to protect neurons from apoptosis.

Genes in Group 2, including PTPRC (CD45), SPP1, and CSF2, showed higher expression levels after treatment with both pramipexole concentrations. It has been observed in mouse models that CD45, upon binding to galectin 1, protects MN9D dopaminergic neurons from MPP + ‐induced damage by reducing the amount of cleaved caspase‐3 and ‐9. However, inhibition of CD45 expression with miRNA reduced the neuroprotective effect.^28^, ^51^ SPP1 (osteopontin), which is expressed in various immune and CNS cells, has shown neuroprotective effects in rat models of ischemia in vivo, by activating the PI3K and P42/44 MAPK pathways.^52^ In addition, it has been observed in mouse models that pretreatment with osteopontin reduces MPTP‐induced damage by switching microglia to an M2 phenotype.^53^

On the other hand, a possible neuroprotective effect has been reported for the CSF2 gene (GM‐CSF), which is also overexpressed. In mouse and rat models treated with MPTP (which overexpresses α‐Syn), administration of lipid nanoparticles containing GM‐CSF mRNA resulted in increased levels of CD4+CD25+Foxp3+ Treg cells, protecting dopaminergic neurons from damage.^54^ A neuroprotective effect has also been reported in MPTP and 6‐OHDA PD models, as BDNF production was increased and the expression of apoptosis‐related proteins, such as Bcl 2 and Bax, was reduced after GM‐CSF administration.^55^

On the other hand, at a dose of 2 ng/mL, pramipexole altered the expression of 34.43% of genes compared with unstimulated cells, whereas 200 ng/mL pramipexole altered 78.69% of genes compared with unstimulated cells. This suggests that 200 ng/mL pramipexole has a greater effect on the transcriptional profile of the cells, which in turn could translate into a greater number of changes in cellular processes. Furthermore, the lower (2 ng/mL) and the higher (200 ng/mL) concentration of pramipexole (Groups 1 and 3 respectively) seem to cause changes in opposite directions: genes selectively upregulated by one concentration of pramipexole are selectively downregulated by the other.

This may indicate that the effect of pramipexole is dose‐dependent. This could be explained by the fact that dopamine receptors are G protein‐coupled receptors (GPCRs) and their activation depends on agonist concentration as a control point for downstream signaling.^56^ It is noteworthy that while genes associated with neuroprotection were found in both Groups 1 and 3, Group 3 contained a greater number of genes with neuroprotective effects, such as GDNF, BDNF, VEGFA, and CD200.

It should be noted that our results are based on an isolated system and that the pathophysiology of PD involves the interaction of several cell populations in addition to neurons and regulatory cells. Nevertheless, our results suggest a possible neuroprotective effect of Tregs. This effect could be enhanced by the presence of other populations, such as antigen‐presenting cells, astrocytes, and microglia, since reduced activation of these populations by immunoregulatory cells has been shown to be beneficial in controlling neuroinflammation.^57^

Finally, our results partially support the use of the dopaminergic agonist pramipexole as a candidate to be administered in combination with L‐dopa, as its effect on regulatory T cells could increase their capacity to protect dopaminergic neurons from damage via IL‐10, which could lead to an improvement in the condition of patients by slowing the progression of the disease.

It is important to recognize the limitations of this study. (1) Regarding the neurite outgrowth experiment, it should be noted that the supernatants used were obtained from co‐culturing IL‐10+ Tregs with neurons. However, supernatants from IL‐10+ Tregs alone were not available in the experiment, and all supernatants were obtained after co‐culture. Although it has been reported that the neurons used on day 28 do not express IL‐10, they do express, albeit in low proportions, two of the subunits of the receptor for this interleukin (IL‐10RA and IL‐10RB).^58^ Therefore, the neurons could be responding to IL‐10 secreted by the Tregs. However, we cannot exclude the possibility that IL‐10+ Tregs produce other molecules that promote neurite outgrowth. (2) Regarding the gene expression experiment, while a select group of genes related to regulation and neuroprotection were included, the complete transcriptome could provide a comprehensive view of all genes and pathways that might be altered after pramipexole administration. Unfortunately, the complete transcriptome of Treg cells is not available. Nevertheless, this is the first study to analyze the effect of pramipexole treatment at the genetic level in human Tregs. Thus, our findings open an important field of study.

## 5 CONCLUSIONS

Our results show that activated Treg cells and IL‐10‐producing, pramipexole‐treated activated Tregs protect dopaminergic neurons from 6‐OHDA‐induced damage. Co‐culture supernatants of activated and dopaminergic agonist‐treated Tregs induced an increase in primary neurite length of dopaminergic neurons, suggesting a promotion of neuronal maturation. In addition, pramipexole‐treated activated Tregs altered their gene expression in a concentration‐dependent manner, including genes previously associated with neuroprotection. Our study sheds new light on the use of dopaminergic agonists in Parkinson's disease and highlights the importance of immunomodulatory cells and treatment in delaying Parkinson's disease progression.

## AUTHOR CONTRIBUTIONS

Adrián Guevara‐Salinas, Diana Denisse Álvarez‐Luquín, and Laura Adalid‐Peralta conceived and supervised the study. Citlalli Netzahualcoyotzi, Edgar Terán‐Dávila, Iván Velasco, and Enrique Estudillo provided stem cells and assisted in the differentiation of dopaminergic neurons. Edgar E. Sevilla‐Reyes assisted in study design/data analysis and provided infrastructure for the development of Fluidigm qPCR chips. Carlos Castellanos‐Barba developed cell purification protocols and provided support in their execution. Adrián Guevara‐Salinas, Citlalli Netzahualcoyotzi, Diana Denisse Álvarez‐Luquín, Erandi Pérez‐Figueroa and Vera Teresa Vega‐Ángeles carried out all the experiments and analyzed the data. Laura Adalid‐Peralta and Adrián Guevara‐Salinas drafted the paper. All authors read and approved the final manuscript.

## FUNDING INFORMATION

This work was supported by Consejo Nacional de Humanidades, Ciencias y Tecnologías (CONAHCYT), Mexico: CONAHCYT FORDECYT‐PRONACES [Ciencia de Frontera No. 2019‐64382] and received funding from PAPIIT‐UNAM (IN219122).

## CONFLICT OF INTEREST STATEMENT

The authors have no conflict of interest to disclose.

## Supporting information


Appendix S1

## Data Availability

The datasets used and/or analyzed during the current study are available from the corresponding author on reasonable request.
