# Supplementary material for: Treating activated regulatory T cells with pramipexole protects human dopaminergic neurons from 6‐OHDA‐induced degeneration
Source: CNS Neurosci Ther. 2024 Aug 4;30(8):e14883. doi: 10.1111/cns.14883 (PMC11298200; doi:10.1111/cns.14883)
Supplement: Supplementary file 1 — Appendix S1 [file CNS-30-e14883-s001.docx]

**SUPPLEMENTARY MATERIALS**

**Supplementary Figure 1.**

**
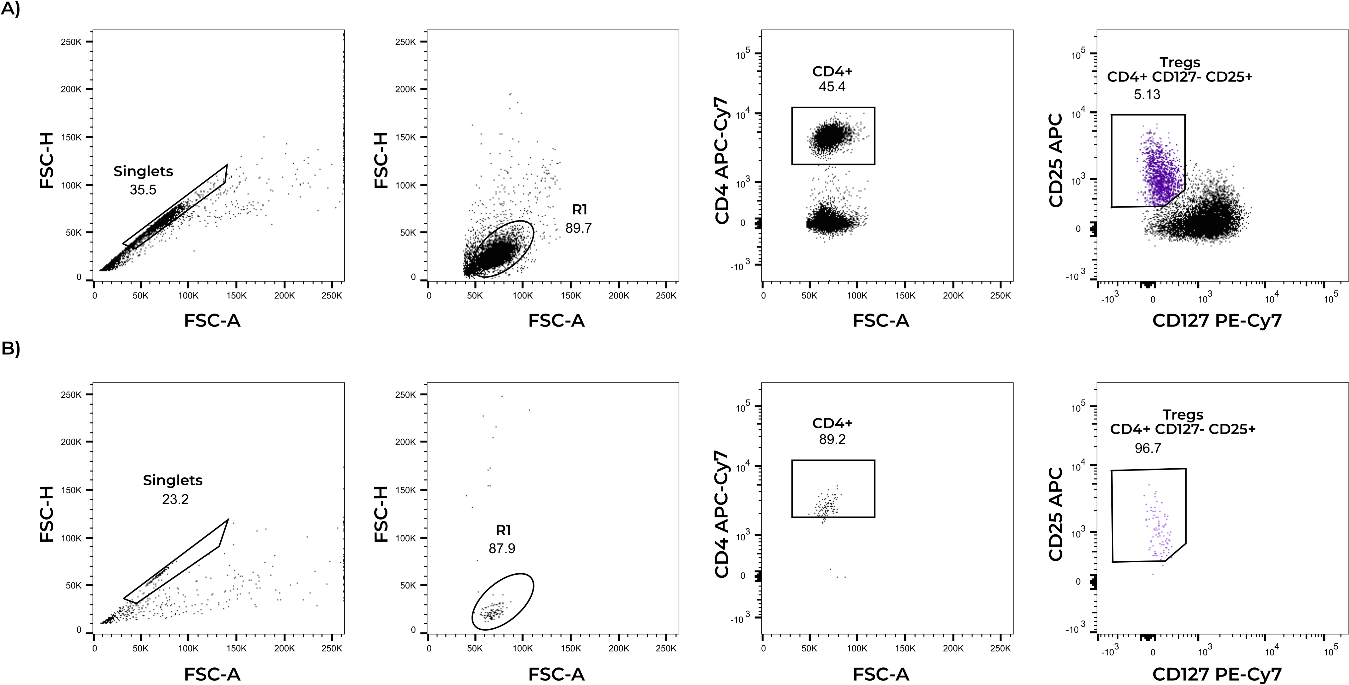
**

**SF1. Gating strategy and purity of Treg cells. A)** Gating strategy for Treg cells. To analyze the Treg (CD4+CD127-CD25+) phenotype, a singlet region was selected; then, the cells were gated according to their forward-side scattering properties; CD4+ cells were selected; then, the gated cells were analyzed for CD25+ and CD127- expression. **B)** Purity of Treg cells after sorting. Cell purity was > 96%.

**Supplementary Figure 2.**

**
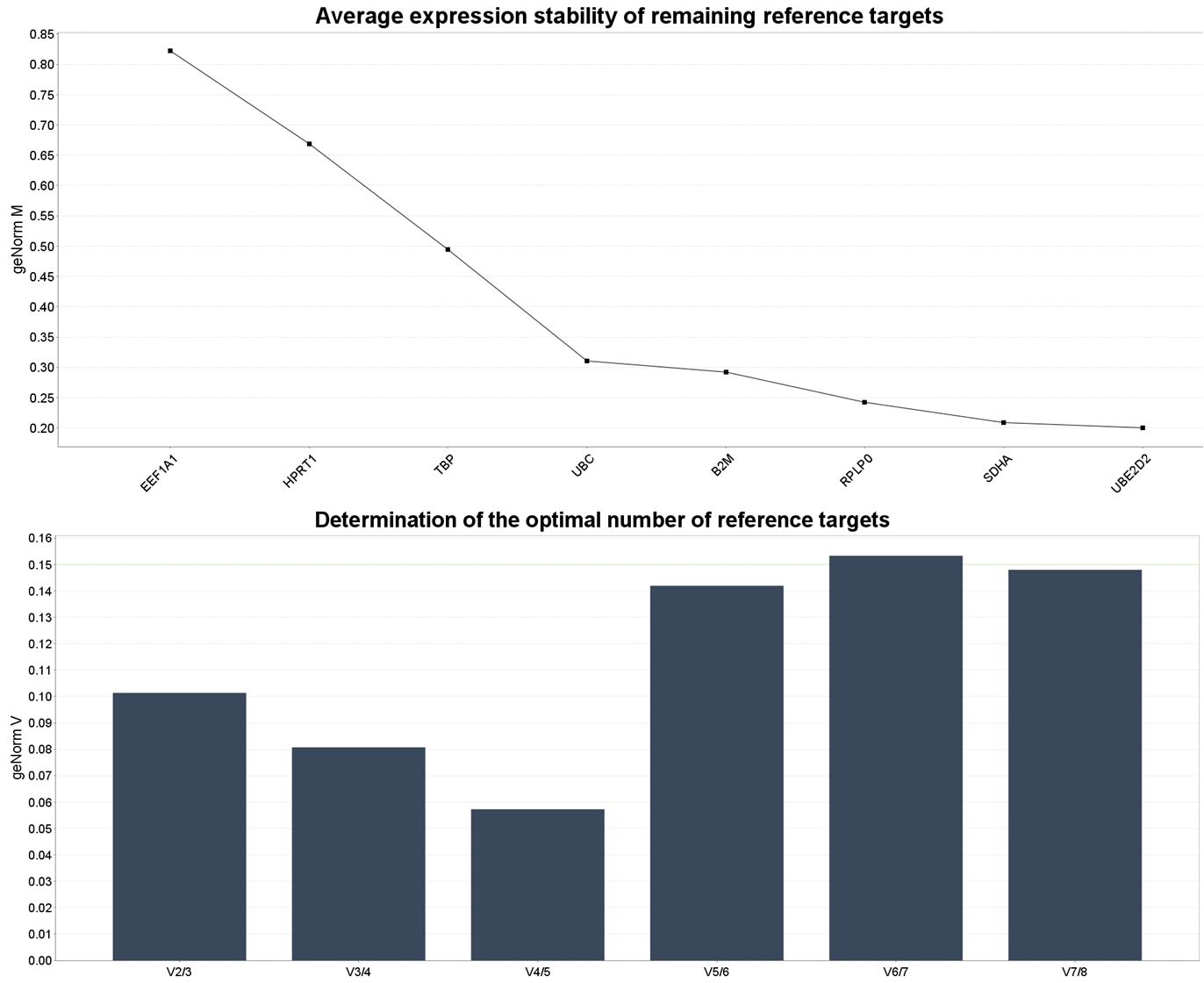
**

**SF2. geNorm V and geNorm M analysis in preactivated Tregs with anti-CD3/CD28.** The most stable reference genes for Tregs preactivated with anti-CD3/CD28 under stimulation conditions were selected according to geNorm V and geNorm M values, prior to data collection and analysis.

**Supplementary Figure 3.**

**
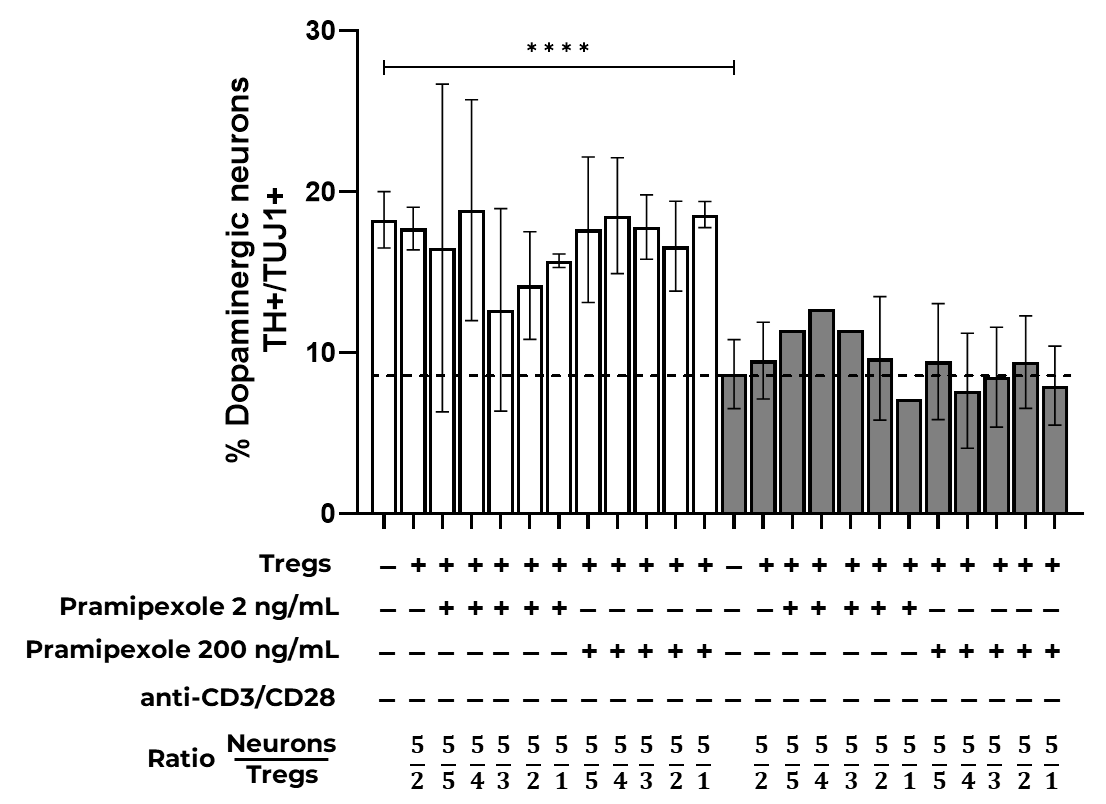
**

**SF3.** **The protective effect of activated Tregs is preserved in different neuron:Treg ratios.** Effect on the percentage of dopaminergic neurons co-cultured with different ratios of pramipexole-treated Treg after 6-OHDA damage. ****p < 0.0001, one-way ANOVA and Dunnett’s post hoc test were used to compare 6-OHDA-treated, control neurons (dotted line) with the other conditions. Bars in all graphs indicate the mean percentage ± SD of at least three independent experiments. White bars indicate control neurons; gray bars indicate 6-OHDA-treated neurons.

**Supplementary Table 1.** A total of 62 human genes were selected based on their involvement in immunoregulatory or neuroprotective processes.

| **Gene Symbol** | **Entrez ID** | **Gene Full Name** |
| --- | --- | --- |
| BDNF | 627 | Brain derived neurotrophic factor |
| BTLA | 151888 | B and T lymphocyte associated |
| CCR2 | 729230 | C-C motif chemokine receptor 2 |
| CCR5 | 1234 | C-C motif chemokine receptor 5 |
| CD200 | 4345 | CD200 molecule |
| CD27 | 939 | CD27 molecule |
| CD274 | 29126 | CD274 molecule |
| CD36 | 948 | CD36 molecule |
| CD38 | 952 | CD38 molecule |
| CD3E | 916 | CD3 epsilon subunit of T-cell receptor complex |
| CD4 | 920 | CD4 molecule |
| CD47 | 961 | CD47 molecule |
| CD5 | 921 | CD5 molecule |
| CSF2 | 1437 | Colony stimulating factor 2 |
| CTLA4 | 1493 | Cytotoxic T-lymphocyte associated protein 4 |
| CX3CR1 | 1524 | C-X3-C motif chemokine receptor 1 |
| DRD1 | 1812 | Dopamine receptor D1 |
| DRD2 | 1813 | Dopamine receptor D2 |
| DRD3 | 1814 | Dopamine receptor D3 |
| DRD4 | 1815 | Dopamine receptor D4 |
| DRD5 | 1816 | Dopamine receptor D5 |
| EBI3 | 10148 | Epstein-Barr virus induced 3 |
| ENTPD1 | 953 | Ectonucleoside triphosphate diphosphohydrolase 1 |
| FASLG | 356 | Fas ligand |
| FOXP3 | 50943 | Forkhead box P3 |
| GDNF | 2668 | Glial cell derived neurotrophic factor |
| GZMB | 3002 | Granzyme B |
| HAVCR1 | 26762 | Hepatitis A virus cellular receptor 1 |
| HAVCR2 | 84868 | Hepatitis A virus cellular receptor 2 |
| GPR81 (HCAR1) | 27198 | Hydroxycarboxylic acid receptor 1 |
| ICOS | 29851 | Inducible T cell costimulator |
| ICOSLG | 23308 | Inducible T cell costimulator ligand |
| IFNG | 3458 | Interferon gamma |
| IL10RB | 3588 | Interleukin 10 receptor subunit beta |
| IL12A | 3592 | Interleukin 12A |
| IL27 | 246778 | interleukin 27 |
| IL2RA | 3559 | Interleukin 2 receptor subunit alpha |
| IL2RB | 3560 | Interleukin 2 receptor subunit beta |
| IL34 | 146433 | Interleukin 34 |
| LAG3 | 3902 | Lymphocyte activating 3 |
| LGALS1 | 3956 | Galectin 1 |
| LGALS3 | 3958 | Galectin 3 |
| LGALS9 | 3965 | galectin 9 |
| NRP1 | 8829 | Neuropilin 1 |
| NT5E | 4907 | 5'-nucleotidase ecto |
| PDCD1 | 5133 | Programmed cell death 1 |
| PDCD1LG2 | 80380 | Programmed cell death 1 ligand 2 |
| PDCD2 | 5134 | Programmed cell death 2 |
| PRF1 | 5551 | Perforin 1 |
| PTPRC | 5788 | Protein tyrosine phosphatase receptor type C |
| SPP1 | 6696 | Secreted phosphoprotein 1 |
| TGFB1 | 7040 | Transforming growth factor beta 1 |
| TGFB3 | 7043 | Transforming growth factor beta 3 |
| TGFBR1 | 7046 | Transforming growth factor beta receptor 1 |
| TGFBR2 | 7048 | Transforming growth factor beta receptor 2 |
| TIGIT | 201633 | T cell immunoreceptor with Ig and ITIM domains |
| TNFRSF18 | 8784 | TNF receptor superfamily member 18 |
| TNFRSF9 | 3604 | TNF receptor superfamily member 9 |
| TNFSF10 | 8743 | TNF superfamily member 10 |
| VEGFA | 7422 | Vascular endothelial growth factor A |
| VIP | 7432 | Vasoactive intestinal peptide |
| B2M | 567 | Beta-2-microglobulin |
| EEF1A1 | 1915 | Eukaryotic translation elongation factor 1 alpha 1 |
| HPRT1 | 3251 | Hypoxanthine phosphoribosyltransferase 1 |
| RPLP0 | 6175 | ribosomal protein lateral stalk subunit P0 |
| SDHA | 6389 | Succinate dehydrogenase complex flavoprotein subunit A |
| TBP | 6908 | TATA-box binding protein |
| UBC | 7316 | Ubiquitin C |
| UBE2D2 | 7322 | Ubiquitin conjugating enzyme E2 D2 |
